# Three-dimensional electron diffraction for porous crystalline materials: structural determination and beyond†

**DOI:** 10.1039/d0sc05731b

**Published:** 2020-12-15

**Authors:** Zhehao Huang, Tom Willhammar, Xiaodong Zou

**Affiliations:** Department of Materials and Environmental Chemistry, Stockholm University Stockholm SE-106 91 Sweden zhehao.huang@mmk.su.se tom.willhammar@mmk.su.se xzou@mmk.su.se

## Abstract

Porous crystalline materials such as zeolites, metal–organic frameworks (MOFs) and covalent organic frameworks (COFs) have attracted great interest due to their well-defined pore structures in molecular dimensions. Knowing the atomic structures of porous materials is crucial for understanding their properties and exploring their applications. Many porous materials are synthesized as polycrystalline powders, which are too small for structure determination by X-ray diffraction. Three-dimensional electron diffraction (3DED) has been developed for studying such materials. In this Minireview, we summarize the recent developments of 3DED methods and demonstrate how 3DED revolutionized structural analysis of zeolites, MOFs, and COFs. Zeolites and MOFs whose structures remained unknown for decades could be solved. New approaches for design and targeted synthesis of novel zeolites could be developed. Moreover, we discuss the advances of structural analysis by 3DED in revealing the unique structural features and properties, such as heteroatom distributions, mixed-metal frameworks, structural flexibility, guest–host interactions, and structure transformation.

## Abstract 2

Three-dimensional electron diffraction is a powerful tool for accurate structure determination of zeolite, MOF, and COF crystals that are too small for X-ray diffraction. By revealing the structural details, the properties of the materials can be understood, and new materials and applications can be designed.

## Graphical abstract

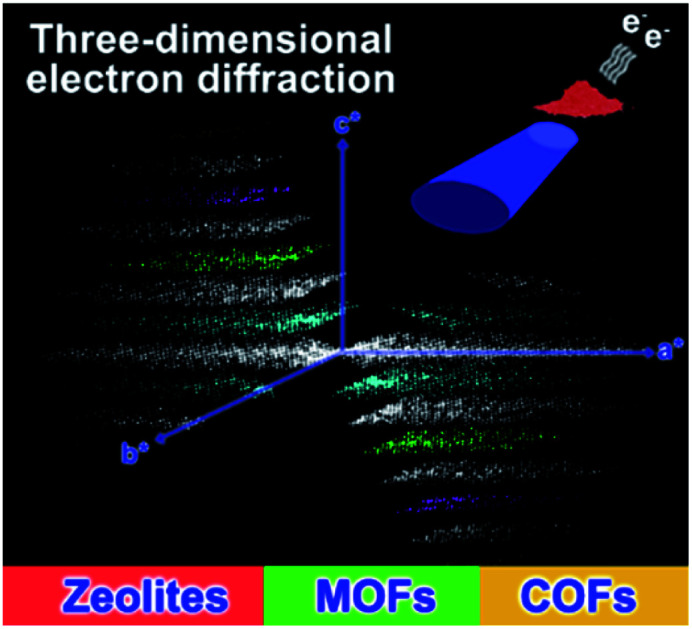

## Introduction

Zeolites,^1,2^ metal–organic frameworks (MOFs),^3,4^ and covalent organic frameworks (COFs)^5^ are among a versatile family of porous crystalline materials. Their physical and chemical properties, such as size and shape selectivity, catalytic activity, conductivity, charge transport, separation ability, *etc.* are closely associated with the underlying atomic structures. These physical and chemical characteristics drive them as attractive materials in a wide range of applications including gas storage and separation, catalysis, energy storage and conversion, sensing, ion-exchange and biomedical applications.^6–9^ To fully implement such materials in practical applications, it is indispensable to understand their physical and chemical properties, which are determined by the arrangement of atoms in the materials. The crystalline nature of zeolites, MOFs, and COFs provides an immense advantage that their structures can be characterized by applying diffraction techniques. Precise and unambiguous structural information can be obtained at atomic levels, in terms of the positions of individual atoms and their connectivities, which is inaccessible to other characterization techniques.

Single crystal X-ray diffraction (SCXRD) is currently the most predominant method for crystal structure determination at the atomic level, but requires large crystals (approx. 5 × 5 × 5 μm^3^ for advanced synchrotron sources) with sufficient quality. Many zeolites, MOFs, and COFs are obtained as nano- or submicron-sized crystals. It is often difficult and sometimes even impossible to grow large crystals, for example, 1D and 2D crystals based on reaction kinetics and thermodynamics. Meanwhile, the sample quantities can be very limited, which prevents testing of enough synthesis conditions for growing large crystals. Although powder X-ray diffraction (PXRD) can be used for structure determination of polycrystalline materials, peak overlap often limits unambiguous indexing and correct intensity estimation of reflections. Structure determination from PXRD becomes more problematic when a crystal has large unit cell dimensions and/or the sample contains multiple phases. During the past two decades, three-dimensional electron diffraction (3DED) techniques,^10,11^ also known as microcrystal electron diffraction (MicroED),^12,13^ have been developed to overcome the barriers for structural analysis of nano- and submicron-sized crystals, which drastically accelerated the development in the fields of zeolites, MOFs, and COFs.

**Scheme 1:**
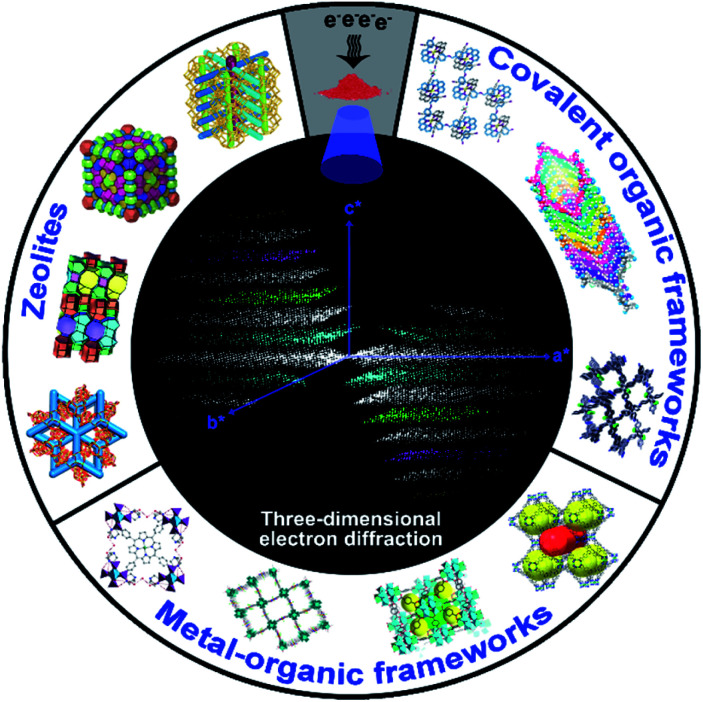
Novel structures and topologies of zeolites, MOFs, and COFs can be determined by using 3DED techniques from nano- and sub-microcrystals, revealing their underlying properties.

In this Minireview, we summarize recent advances in the development of 3DED methods, and their applications for structural analysis of zeolites, MOFs, and COFs, with emphasis on novel materials (Scheme 1). We describe how the detailed atomic structures obtained by 3DED can reveal the key structural features, uncover structure–property relationships, and provide an increased understanding of the properties of such materials. We believe that a timely critical overview on 3DED is of great importance to advance our understanding towards the development of porous crystalline materials, where large crystals are no longer mandatory for single crystal structural analysis.

## Three-dimensional electron diffraction (3DED)

In combination with imaging techniques, electron diffraction (ED) taken along well-defined zone axes had been applied for structure determination of nano- and submicron-sized crystals for more than 50 years.^14–22^ However, collection and processing of such in-zone ED data for 3D structure determination is both demanding and time-consuming, requiring strong electron microscopy and crystallography expertise. More importantly, ED intensities suffer from dynamical effects generated by multiple scattering events of electrons in the crystal. These have hampered whispered application of electron diffraction though examples of using it for *ab initio* structure determination have been reported.^16^

**Fig. 1:**
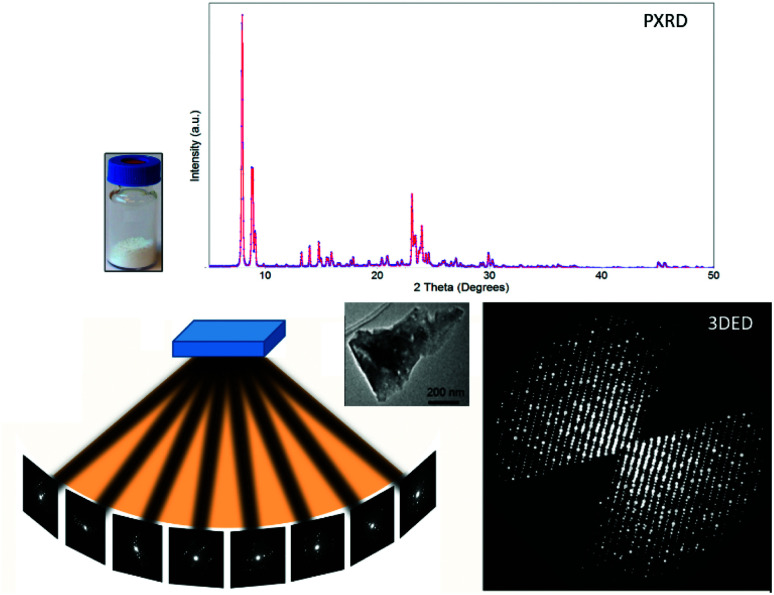
Comparison of PXRD and 3DED data obtained from polycrystalline samples. PXRD patterns are collected from millions of crystallites but contain a limited number of peaks due to overlapping. Meanwhile, 3DED data are collected from different orientations of a single crystal, and provide thousands of diffraction peaks.^31^ 3DED data contain a missing wedge due to the rotation angle limited by the TEM while PXRD patterns are complete. Reproduced from ref. 31 with permission from the Elsevier, copyright 2014.

The revolution in electron crystallography started in late 2000s, when Kolb *et al.* and Hovmöller *et al.* independently developed software to control three-dimensional electron diffraction (3DED) data collection (Fig. 1). This is achieved by stepwise rotation of a crystal along an arbitrary axis and collecting ED patterns at each angle. They applied different approaches to achieve fine sampling of reciprocal lattices and integrated intensities. Kolb *et al.* developed automated diffraction tomography (ADT)^10^ to control the goniometer tilt and applied beam precession using dedicated hardware.^23^ Hovmöller *et al.* developed rotation electron diffraction (RED) software to control both the goniometer and the electron beam, combining coarse crystal rotation with fine beam tilt^11,24^ (Table 1). They have also developed software to process the ED patterns from different angles and reconstruct 3D reciprocal space (Movie S1†). This new development of 3DED no longer requires alignment of crystals at a zone-axis, which both simplifies and speeds up the data collection. More importantly, dynamical effects are reduced when crystals are oriented off zone axes. As a result, intensities obtained from 3DED are less dynamical and in most cases can be treated as kinematical intensities, so that similar routines and software for structure determination by SCXRD can be applied on 3DED data (Fig. 1).^25^

**Table 1:** List of 3DED protocols

Protocol name	Data collection strategy	Data collection speed	EM mode	Beam tilt	Beam procession	Publication year	Reference
ADT/PEDT	Stepwise	Slow	STEM	No	Yes	2007	10 and 26
RED	Stepwise	Slow	TEM	Yes	No	2010	11 and 24
EDT	Stepwise	Slow	TEM	Yes	No	2013	27
MicroED	Stepwise	Slow	TEM	No	No	2013	12
Rotation electron diffraction	Continuous	Fast	TEM	No	No	2013	28
MicroED	Continuous	Fast	TEM	No	No	2014	13
Fast EDT	Continuous	Fast	TEM	No	No	2015	29
cRED	Continuous	Fast	TEM	No	No	2018	30

Porous materials often suffer from electron beam damage, which diminishes the crystallinity during data acquisition. This drawback has been overcome with the development of continuous rotation data collection methods,^13,28–30^ another milestone in the development of 3DED. In contrast to stepwise methods, the crystal rotates continuously during the entire data collection, and ED patterns are simultaneously recorded as a movie. Because the crystals can move during rotation, Cichocka *et al.* developed a crystal-tracking solution to ensure that the same crystal area is used during the data acquisition.^30^ By adjusting the goniometer rotation speed and using a fast detector, a full 3DED dataset can be acquired very fast, within one minute (Movie S2†). The short data collection time combined with a low electron dose rate (<0.1 e s^−1^ Å^−2^) can effectively reduce the total electron dose applied on the analytes, and thus minimizes beam damage. Notably, because intensities are integrated on each ED pattern by using the continuous rotation methods, more accurate intensities can be obtained compared to step-wise rotation. Several groups developed their own protocols for continuous rotation electron diffraction data collection, including microcrystal electron diffraction (MicroED),^13^ fast electron diffraction tomography (Fast EDT^29^), rotation electron diffraction^28^ and continuous rotation electron diffraction (cRED)^30^ (Table 1). By applying 3DED, thousands of diffraction peaks can be obtained from a single nanocrystal, while only a limited number of peaks are observed in a PXRD pattern (Fig. 1). The high number of datasets ensures an unambiguous determination of unit cells and space groups. Moreover, it allows reliable structure solution and refinement despite the presence of dynamical effects.

To process 3DED data, several programs, such as RED data processing,^24^ ADT 3D,^32^ and PETS^33^ have been developed. Software developed and widely used for X-ray crystallography, such as XDS^34^ and DIALS,^35^ can be easily adapted for processing continuous rotation electron diffraction data. After extracting the intensities, structure solution and refinement are performed by applying similar approaches to those for SCXRD, using direct methods (SHELX,^36^ SIR^37^) or charge flipping (JANA^38^). These programs treat intensities from 3DED as kinematical. Atomic scattering factors for electrons are used instead of those for X-rays. Various structures of porous crystalline materials, which could not be solved by X-ray diffraction, have now been determined by 3DED, and the number keeps growing.

The strong interactions between electrons and matter allow obtaining high-resolution 3DED data from nano- and submicron-sized crystals. On the other hand, the strong interactions also lead to multiple dynamical scattering events that cause the intensities of electron diffraction to deviate from kinematical intensities. Although dynamical effects can be reduced by 3DED techniques where data are collected at arbitrary off-zone orientations, structure refinements against 3DED data still lead to relatively high *R*_1_ values, which indicate the difference between the intensities calclated from the model and the observed intensities. This has been a key concern in the field of electron crystallography. Nevertheless, recently studies on zeolites^39^ and MOFs^40^ have proved that the structural models obtained from 3DED are similar to those obtained by SCXRD, with average deviations of atomic positions by less than 0.10 Å. Moreover, structure determination by 3DED also shows high reproducibility. The deviations of atomic positions obtained from 3DED data of different individual crystals are less than 0.10 Å.^39,41^ The high accuracy is attributed to the high ratio (usually >10) of the number of reflections to the number of refined parameters. Despite the high accuracy, however, because the structure refinement software used is developed for SCXRD, which assumes the intensities to be kinematical. Thus, the resulting high *R*_1_ values mainly show that the observed 3DED intensities are dynamical and deviate from kinematical intensities calculated from the model. To take dynamical effects into account and further improve the structure refinement, Palatinus *et al.* developed dynamical refinement^42^ that compares observed PEDT intensities with dynamical intensities. They show that more accurate structural models including hydrogen positions could be obtained, and *R*_1_ values were greatly reduced.

**Fig. 2:**
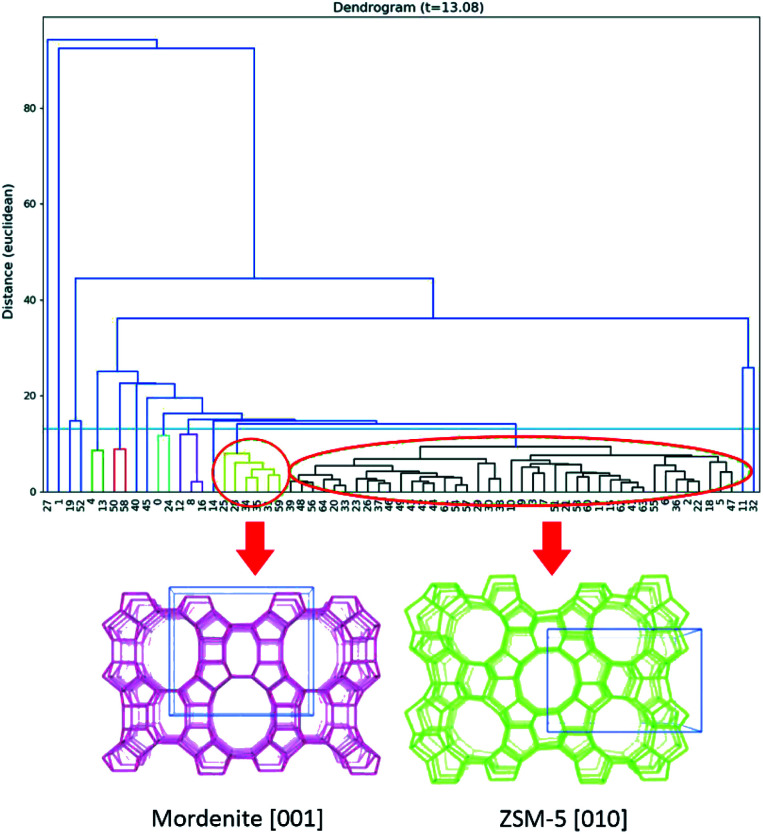
Dendrogram of the hierarchical cluster analysis (HCA) based on unit cells. Two clusters are identified, corresponding to mordenite and ZSM-5, shown as yellow and black clusters respectively (marked by circles).^43^ Reproduced from ref. 43 with permission from the International Union of Crystallography, copyright 2019.

An important advantage of 3DED is that each dataset can be collected in a few minutes. Thus, it is possible to collect datasets from many different individual crystals. The different datasets can be merged to overcome the missing-wedge problem and achieve high data completeness. Based on this, a new approach, serial rotation electron diffraction (SerialRED), has recently been developed to achieve fully automated crystal screening and cRED data collection.^43^ It is capable of screening up to 500 crystals per hour. A data processing pipeline using hierarchical cluster analysis (HCA) was developed to automatically process each dataset, group the phases present in the sample and find the best matching datasets for subsequent data merging and structural analysis (Fig. 2). This method enables high-throughput phase analysis and structure determination, which is especially powerful for studies of porous crystalline materials.

## Application of 3DED to zeolites

Zeolites, including aluminosilicates, germanosilicates, borosilicates, aluminophosphates, *etc.*, are crystalline materials built from corner-sharing TO_4_ tetrahedra. Zeolite frameworks have well-defined pores and channels, which are important for catalysis and separation. Prior to the development of 3DED, electron microscopy, especially high-resolution transmission electron microscopy (HRTEM) imaging, has played a crucial role in structural studies of zeolites. The readers are referred to recent review articles on HRTEM imaging of zeolites.^17,22^ Compared to HRTEM imaging, 3DED is more feasible and less demanding in terms of both instrumentation and expertise. During the past decade, 3DED has played a dominating role in the discovery of novel zeolites and determination of their structures. Among the 56 new zeolite framework types approved by the Structure Commission of the International Zeolite Association (IZA) since 2011,^44^ 32 have been discovered using 3DED (Table 2).

**Table 2:** List of novel zeolite structures discovered using 3DED^44^

Name	Code	Channel dimension and sizes	3DED method	Publication year	Name	Code	Channel dimension and sizes	3DED method	Publication year
ITQ-43		3D, 28 × 12 × 12	ADT	2011	SCM-14	SOR	3D, 12 × 10 × 10	RED	2017
ITQ-51	IFO	1D, 16	RED	2013	ZSM-43	MRT	2D, 8 × 8	RED	2017
EMM-23^a^	-EWT^a^	3D, 24 × 10 × 10	RED	2014	IM-18^a^	UOE^a^	1D, 10	RED	2018
ERS-18	EEI	2D, 8 × 8	RED	2014	PST-13	POR	3D 8×	cRED	2018
IM-17	UOV	3D, 12 × 10 × 8	ADT	2014	PST-14				
PKU-16	POS	3D, 12 × 11 × 11	RED	2014	SYSU-3	-SYT	3D, 24 × 8 × 8	cRED	2018
PST-6	PSI	1D, 10	RED	2014	ECNU-21	EWO	1D, 10	cRED	2019
CIT-7	CSV	2D, 10 × 8	RED	2015	EMM-37	ETV	3D, 10 × 8 × 8	cRED	2019
ITQ-53	-IFT	3D, 14 × 14 × 14	RED	2015	SCM-15		3D, 12 × 12 × 10	cRED	2019
ITQ-54	-IFU	3D, 20 × 14 × 12	RED	2015	SSZ-27		1D, 8	cRED	2019
PST-20		3D, 8 × 8 × 8	RED	2015	ECNU-23		2D, 12 × 8	cRED	2020
ZSM-25	MWF	3D, 8 × 8 × 8	RED	2015	IDM-1^a^		3D, 17 × 17 × 10	cRED	2020
CIT-13^a^	CTH^a^	2D, 14 × 10	RED	2016	PST-5		2D, 10 × 8	cRED	2020
EMM-26	EWS	2D, 10 × 10	RED	2016	PST-24^a^		2D, 10 × 10	cRED	2020
							3D, 10 × 10 × 10		
ITQ-58		2D, 8 × 8	Fast-EDT	2016	RUB-5^a^		2D, 8 × 8	ADT	2020

a Disordered structures.

### Extra-large pore zeolites

The pore openings of zeolites are classified by the number of TO_4_ tetrahedra, as small (8-ring), medium (10-ring), large (12-ring) and extra-large (≥14-ring) pores. Currently, only 23 extra-large pore zeolites have been reported.^44^ Among the 10 extra-large pore structures reported in the past 10 years, eight (ITQ-43,^45^ ITQ-53,^46^ ITQ-54,^47^ CIT-13,^48^ SYSU-3,^49^ ITQ-51,^50^ EMM-23 ^51^ and IDM-1 ^52^) were determined by 3DED. ITQ-43 with extra-large 28-ring channels was the first example of *ab initio* structure determination of zeolites by 3DED.^45^ The structure was solved by direct methods using SIR2008, from ADT data collected at 100 K. All 20 Ge/Si and 13 out of 24 oxygen positions were directly located. The missing oxygen atoms were either found from difference Fourier maps or placed according to the geometry. It is common that extra-large pore zeolites have interrupted frameworks with terminal T-OH groups. The terminal groups can be further disordered, such as in EMM-23,^51^ and IDM-1.^52^ The average structures of EMM-23 and IDM-1 were also determined by 3DED. In these cases, solid-state NMR played a complementary role in determining the disordered terminals in the framework. Disorder can be utilized to alter the pore sizes. For instance, the channel size of EMM-23 can be varied from 21- to 24-ring channels by tuning the occupancy of one terminal T-site from one to zero.

### Improvement of data quality for beam sensitive zeolites

Many zeolites are sensitive to the electron beam, and hence it is advantageous to use low electron dose and fast data collection for acquiring high-resolution 3DED data. For this purpose, Simancas *et al.* applied Fast-EDT data collection that combines precession-assisted electron diffraction tomography (PEDT) with fast continuous goniometer rotation speed (1.67° s^−1^) for *ab initio* structure determination of a borosilicate ITQ-58.^53^ The Fast-EDT data were collected using a GATAN Orius SC600A CCD camera. A dataset could be collected within 30 seconds, with a high data resolution (0.88 Å). Because of the low symmetry (triclinic crystal system) and limited goniometer rotation range (*ca.* 50°), each dataset had only a completeness of *ca.* 30%, which was too low for structure solution by direct methods. Four datasets from different crystals were then merged to achieve a higher data completeness of 41%. The structure of ITQ-58 could be solved and refined from the merged dataset. Due to the low data completeness, restraints were applied in the refinement, and the same atomic displacement parameters (ADPs) were applied to each atom type.

Using a hybrid pixel detector with fast data readout, fast cRED data collection (0.5–3 min per dataset) can be achieved. Combined with the crystal tracking system, it enables collecting multiple datasets over a large angular range to reach a high data completeness. The aluminosilicate EMM-37 also crystallizes in a triclinic system.^54^ Using cRED, the merged dataset reached a completeness of 79.8% with a resolution of 0.70 Å, allowing refinement of anisotropic ADP of each individual atom without applying any restraints. cRED revealed that EMM-37 has a 3D 10- and 8-ring channel system. Notably, the pore widows became narrowed after calcination. This property is crucial for EMM-37 to selectively separate ethane over propane.

### Heteroatom distribution in zeolites

**Fig. 3:**
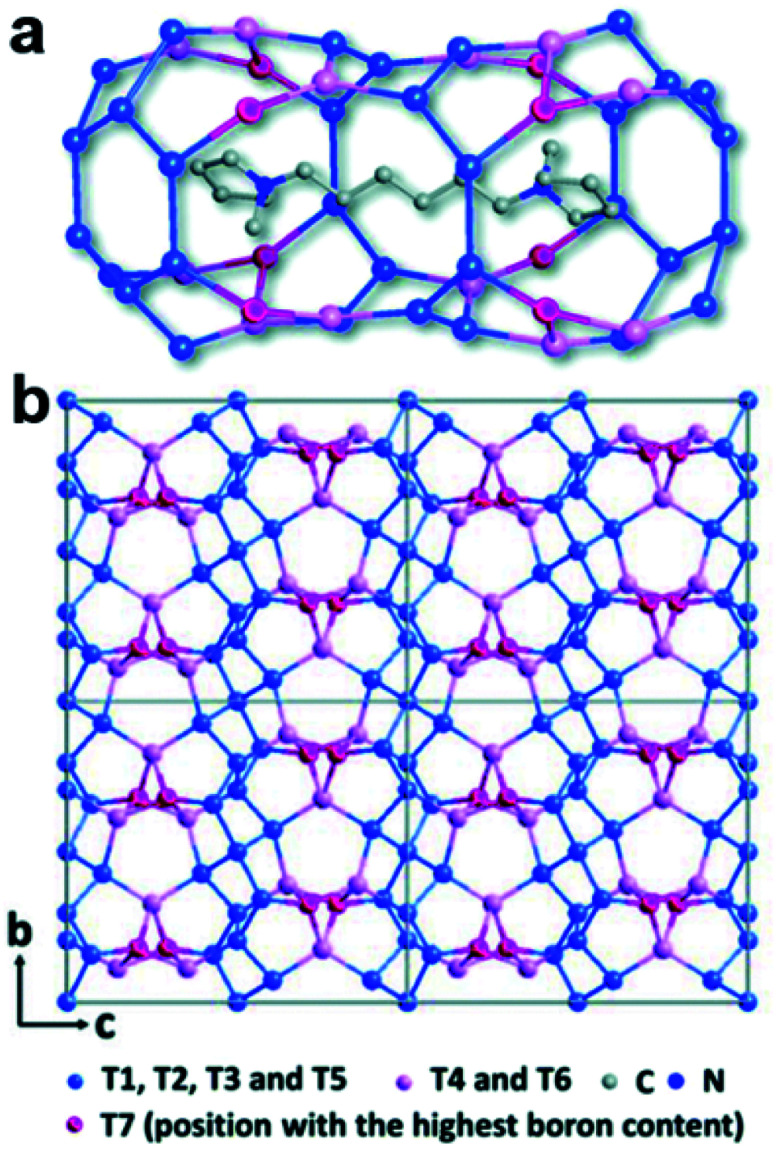
The structural model of EMM-26. (a) The cavity in EMM-26 where the organic template resides. (b) The structural model viewed along the [100] direction. The Si-rich sites are shown in blue and B-rich sites in pink and magenta. Bridging O atoms have been omitted for clarity.^55^ Reproduced from ref. 55 with permission from the Royal Chemical Society, copyright 2016.

A well-developed strategy to obtain novel zeolite structures is to introduce main block elements such as B, Ge, *etc.* in the frameworks. It is important to find out the location of these elements in the frameworks because this knowledge is important to fundamentally understand the synthesis and the properties of the materials. The structure of a novel borosilicate zeolite EMM-26 was determined by RED,^55^ which comprises two-dimensional (2D) intersecting 10 × 10-ring channels (Fig. 3). Notably, the occupancies of Si and B atoms could be revealed from the refinement against RED data, showing that B atoms preferentially occupied three of the seven unique T sites, with the occupancy of 12%, 5%, and 39%, respectively. This agrees well with those of the corresponding sites (14%, 8% and 35%, respectively) refined against PXRD data.

### Towards targeted zeolite synthesis

**Fig. 4:**
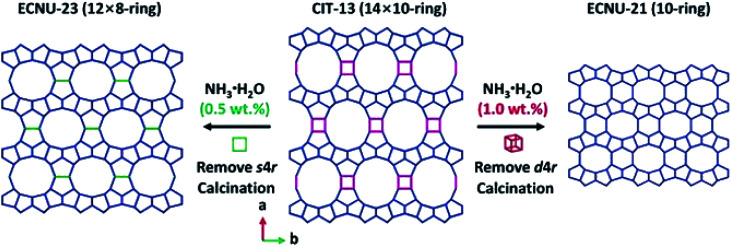
Structural transformation from germanosilicate CIT-13 into high silica zeolites ECNU-21 and ECNU-23.^57^ The structures of all three zeolites were discovered by cRED. Reproduced from ref. 57 with permission from the John Wiley & Sons, Inc., copyright 2020.

ADOR (Assembly–Disassembly–Organization–Reassembly) is one of the strategies for targeted synthesis of novel zeolites.^56^ By selectively breaking and removing the weak Ge(Si)–O–Ge chemical bonds within the d4r units of the germanosilicate CIT-13 (UTL, 14 × 10-ring), two new zeolites, ECNU-21 (12 × 8-ring) and ECNU-23 (10-ring), were prepared. The structures of all three zeolites were determined by cRED (Fig. 4).^48,57^ Among them, ECNU-23 was first discovered as an “impurity” from a so-called “pure” phase of ECNU-21 as determined by PXRD. After revealing the new ECNU-23 structure by cRED, synthesis conditions were optimized to obtain the phase pure ECNU-23 material.^57^ The unique benefit to individually characterize each submicron-sized crystal by cRED enables studies of structural heterogeneities within a sample. This marks an unprecedented advantage to discover novel materials formed as a minor impurity. The germanosilicate PKU-16, which has a 3D 12 × 11 × 11 channel system, is another example of this kind.^58^ The structure of aluminosilicate SSZ-27 was also determined from a sample containing impurity. Among the cRED data collected on 18 crystals, 14 were from SSZ-27 and the remaining four were from the known zeolite SSZ-26 (CON). Hierarchical cluster analysis was used to identify the most consistent datasets to be merged prior to structure refinement in order to increase the data completeness.^59^

**Fig. 5:**
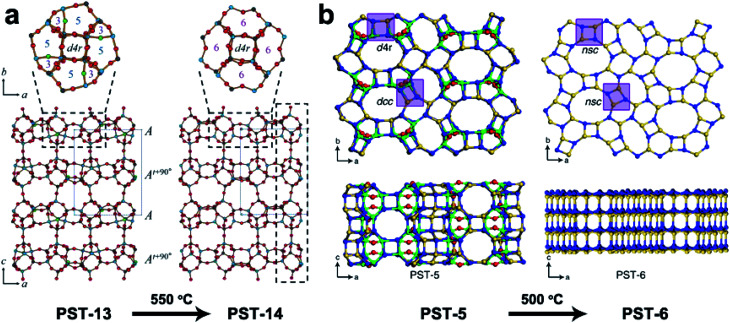
Structural transformation of the aluminophosphates PST-13 to PST-14 (a) and PST-5 to PST-6 (b) by dehydration. While the transformation from PST-13 to PST-14 only involves the loss of bridging –OH groups and no change in the framework topology,^62^ that from PST-5 to PST-6 involves transformation of double-crankshaft chain (*dcc*) and double four ring (*d*4*r*) units to narsarsukite-type chain (*nsc*) units. This leads to topologically different framework structures. The pore system of the material changes from the 2D channel system of PST-5 to a 1D system.^60^ Reproduced from ref. 60 and 62 with permission from the John Wiley & Sons, Inc., and Springer Nature, respectively, copyright 2018 and 2020.

3DED has also been used for *ab initio* structure determination of (silico)aluminophosphates such as ITQ-51,^50^ PST-5,^60^ PST-6,^61^ PST-13 ^62^ and PST-14.^62^ PST-5 and PST-13 were synthesized using diethylamine (DEA) as the organic structure directing agent (OSDA). Both structures contain penta-coordinated framework Al atoms connected by OH groups, which could be removed by calcination to form fully tetra-coordinated frameworks PST-6 and PST-14, respectively (Fig. 5). Interestingly, while the framework topology of PST-14 remained the same as that of PST-13, the framework topology of PST-5 changed completely upon dehydration. It is revealed by cRED that the structural changes between PST-5 and PST-6 involve a 3D–3D topotactic transformation between the *dcc*/*d*4*r* and *nsc*. Based on the structural transformations observed in PST-5/PST-6, AlPO-21/AlPO-25 and AlPO-C/AlPO-D pairs, a new approach to generate novel structures in aluminophosphate systems was proposed. A series of new synthetically feasible zeolite structures were predicted.^60^ This approach could enable targeted synthesis of novel phosphate-based materials, which adds to the existing ADOR approach applicable on silicate-based zeolites.

### Decades-long puzzles in zeolite structures

**Fig. 6:**
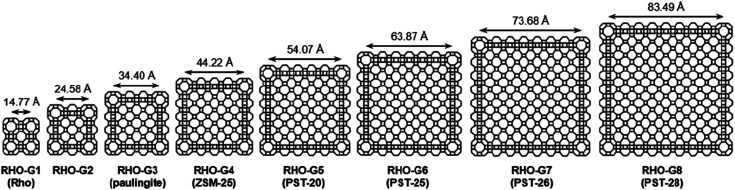
Framework representations of cross sections (*ca.* 12 Å thick) of RHO-G1 to RHO-G8 in the RHO family of embedded isoreticular zeolites.^66^ Reproduced from ref. 66 with permission from the John Wiley & Sons, Inc., copyright 2016.

In addition to structure determination of novel zeolites, 3DED has also made it possible to determine structures of zeolites that remained unsolved for decades. For example, the structures of two aluminosilicates ZSM-43 ^63^ and ZSM-25,^64^ and a 2D natural zeolite cowlesite^65^ were solved from 3DED data after remaining elusive for more than 30 years. The structures of ZSM-43 and cowlesite were solved from high-resolution (∼1.0 Å) data by direct methods. However, the RED data from ZSM-25 had a low resolution (2.5 Å) due to beam damage. The data were sufficient for the determination of the unit cell and space group, but not enough for *ab initio* structure solution. Fortunately, two structures in the IZA Database of Zeolite Structures,^44^ namely rho (RHO) and paulingite (PAU) were found to be closely related to ZSM-25. All three have the same space group and their unit cell parameters differ by 20 and 10 Å, respectively. More importantly they show a similar intensity distribution of reflections in reciprocal space, indicating that they belong to the same RHO family. The structure of ZSM-25 was thus solved using the strong reflections, with their intensities from RED, and the corresponding crystallographic structure factor phases from PAU. The structural details of ZSM-25 revealed by 3DED opened up an isoreticular route for targeted synthesis of new zeolites. By adopting this new strategy, five new embedded isoreticualr zeoliltes in the RHO family were synthesized (Fig. 6).^64,66,67^

### Disordered zeolites

**Fig. 7:**
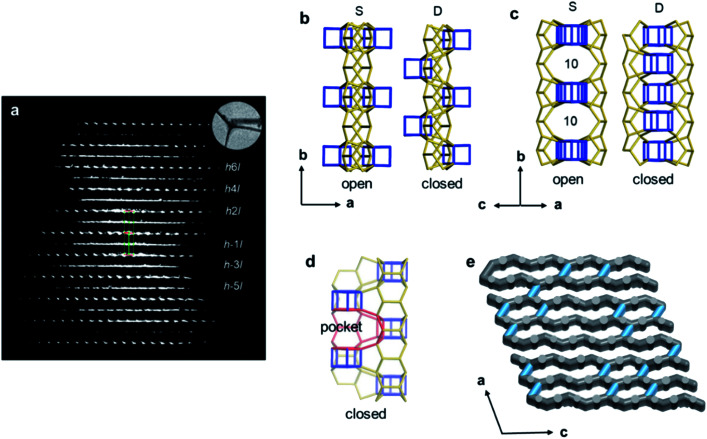
(a) Reconstructed reciprocal lattice from a crystal of PST-24. Viewing along (b) [001] and (c) [101] directions, different arrangements of the *d*5*r* units (in blue) result in open or closed 10-ring channels. (d) The pockets, which are formed instead of the additional channels, are observable, and one of them is marked in red. (e) One hypothetical model of the channel system of PST-24. The 10-ring channels along the [101] direction are randomly distributed (shown in light blue).^70^ Reproduced from ref. 70 with permission from the John Wiley & Sons, Inc., copyright 2020.

Structural disorders are an obstacle in structure determination, particularly for zeolites due to their structural complexity. 3DED has been proved as a powerful tool for obtaining the average structures. Yet, other characterization techniques, such as HRTEM imaging, could be valuable and provide additional structural information as demonstrated for ITQ-39 ^68^ and SSZ-61.^69^ PST-24 is one example, whose average structure was determined *ab initio* by cRED.^70^ The reconstructed 3D reciprocal lattice of PST-24 is featured by alternating rows of sharp spots and diffuse lines (Fig. 7a), indicating the presence of disorder. Nevertheless, from the cRED data, the average unit cell could be determined to be *a* = 24.14(4) Å, *b* = 5.21(2) Å, *c* = 21.76(3) Å, *α* = 90°, *β* = 111.4(2)°, *γ* = 90°. The possible space groups were deduced as *C*2, *Cm*, and *C*2/*m*. Based on this structural information, intensities were extracted from the sharp Bragg reflections, from which an average structure was obtained directly. The revealed structure is composed of cas-zigzag chains and *d*5*r* units. However, the *d*5*r* units in the average structure are so close to each other that only every second *d*5*r* units in the column along *b*-axis can exist simultaneously. This resulted in disorders in PST-24 with either open or closed 10-ring channels (Fig. 7b and c), and a channel system varying locally from 2D to 3D (Fig. 7e). HRTEM imaging further shows that PST-24 crystals are 1D ordered and 2D disordered. A similar 3DED approach was used to determine the average structure of the disordered silicogermanate IM-18 (*UOE), with HRTEM imaging revealing the locations and domain sizes of the multidimensional stacking disorders.^71^ Furthermore, 3DED was also used to quantify stacking disorder in zeolite Beta.^72^ It is important to note that compared to HRTEM imaging, 3DED data acquisition is simpler and requires much lower electron doses. In addition, 3DED can be performed on a standard TEM and there is no need to align the crystals. On the other hand, HRTEM imaging gives additional information about local structural details, and is complementary to 3DED for studying disordered materials.

## Application of 3DED to MOFs

SCXRD has been the most widely used technique for determination of MOF structures. Yet, many MOFs crystallize as nano- or submicron-sized crystals, which are too small for SCXRD. Thus, 3DED has become an important technique for structure determination of such MOF crystals. Due to the labile coordination bonding between metals and organic linkers, MOFs suffer more from electron beam damage compared to zeolites. Therefore, during the early applications of 3DED techniques, the data resolution was limited, and most structures could only be solved by model building based on the unit cell and space group obtained by 3DED. Recent evolution of 3DED through continuous rotation methods has overcome the challenge of beam damage and greatly improved the data quality. Therefore, it is much easier to perform *ab initio* structure determination on nano- and submicron-sized MOFs nowadays.^40,41,73–85^ The revealed structural details provide an enhanced understanding of their properties as well as fundamental knowledge to promote the discovery of new materials.

### *Ab initio* structure determination of MOFs

**Fig. 8:**
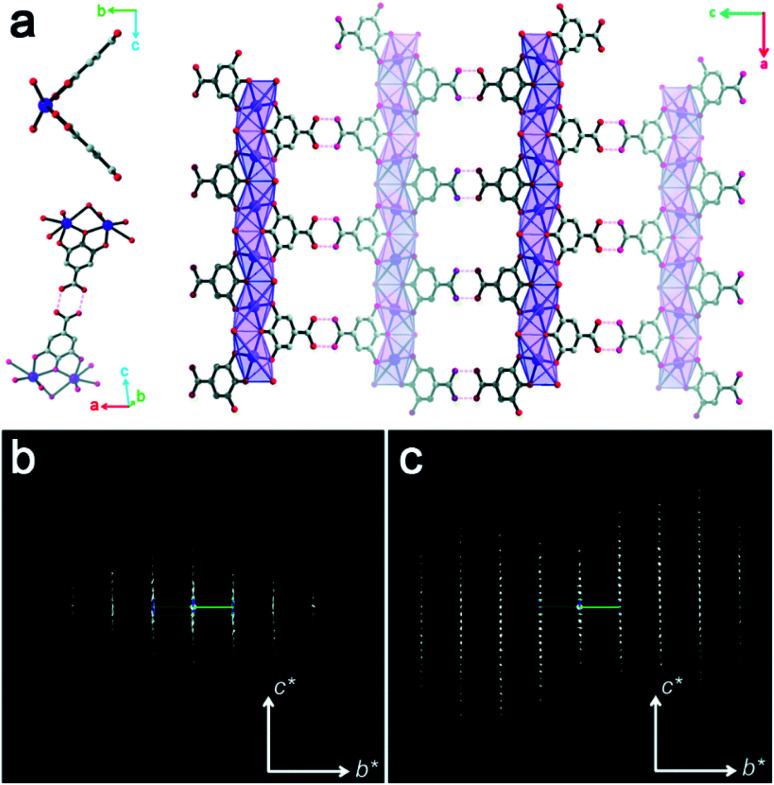
(a) Structural model of bismuth subgallate, showing a one-dimensional structure with the framework stabilized by intermolecular H-bonding. The 3DED data collected by (b) stepwise RED and (c) cRED with sample cooling, showing a significant improvement on data resolution.^75^ Reproduced from ref. 75 with permission from the Royal Chemical Society, copyright 2017.

The first example of *ab initio* structure determination of a MOF nanocrystal was MFU-4l, using ADT data with 1.3 Å resolution. MFU-4l was shown to have a cubic six-connected net by linking bis(1*H*-1,2,3-triazolo[4,5-*b*],-[4′,5′-*i*]dibenzo[1,4]dioxin) (BTDD^2−^) with the pentanuclear (Zn_5_Cl_4_)^6+^ clusters.^73^ Another example was the discovery of the structure of a century-long-used pharmaceutical ingredient bismuth subgallate.^75^ Bismuth subgallate was previously believed to be a complex between Bi^3+^ and gallate anions, with the gallate latching on *via* its carboxylate group. The structure solution from cRED data shows that bismuth subgallate is in fact a one-dimensional coordination polymer where the gallate anions coordinate *via* the phenolates rather than carboxylate groups (Fig. 8a). Notably, as bismuth subgallate is sensitive to electron beam, sample-cooling and fast data collection were crucial, which improved the data resolution from 1.5 Å to 1.0 Å (Fig. 8b and c). Another MOF with potential bio-related applications, sc-CCMOF-1 made by Zn(ii) cations and curcumin, was characterized using EDT data with a resolution of 1.0 Å and completeness of 0.94. sc-CCMOF-1 was proved to be a permanently porous MOF.^77^

**Fig. 9:**
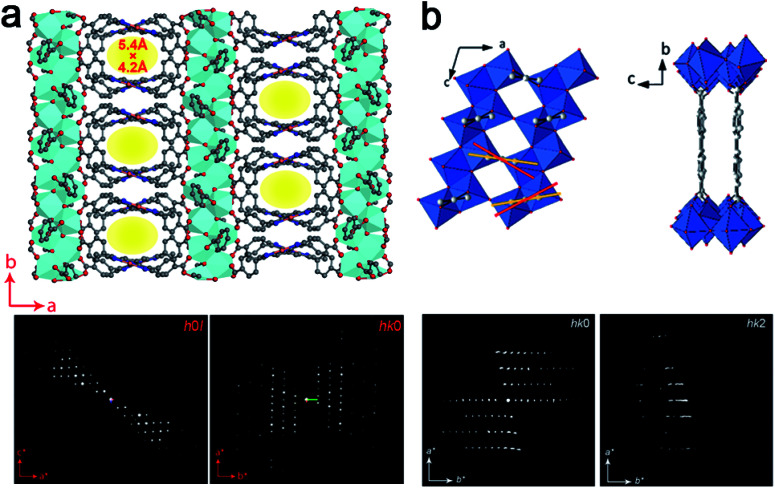
Structural models and corresponding cRED data of (a) PCN-226, a Zr-oxo chain based structure,^84^ and (b) COK-47, a Ti-oxo sheet based structure.^82^ All of them are obtained as nanocrystals, and the structures are determined using cRED data. Reproduced from ref. 82 and 84 with permission from the American Chemical Society, and John Wiley & Sons, Inc., respectively, copyright 2020 and 2019.

Notably, 3DED is particularly powerful for determining unexpected novel structures. For example, cRED was used to determine the structure of PCN-226, which was crystallized in nano-size.^84^ From cRED data with 1.15 Å resolution, PCN-226 was revealed to contain rare Zr-oxo chains connected by tetrakis(4-carboxyphenyl)porphyrin (TCPP) ligands (Fig. 9a). The unique chain-based structure revealed by cRED showed an unexpected new topology, which was utilized as a favorable system for electrochemical applications. COK-47 is another novel nanocrystalline MOF determined from *ab initio* using cRED data.^82^ Its structure is built from Ti-oxo sheets linked together by 4,4′-biphenyldicarboxylate (bpdc^2−^) anions (Fig. 9b).

As aforementioned in zeolite materials, 3DED can be used to determine preferred locations of different metals based on intensities of electrostatic potentials and coordination environments. This is particularly important for mixed-metal MOFs, where the positions of different metal cations play important roles in their physical and chemical properties. For example, the Ti(iv) and Zr(iv) cations as well as the linkers in PCN-415 could be located directly by *ab initio* structure determination using cRED data, which show that Ti(iv) and Zr(iv) cations form bimetallic [Ti_8_Zr_2_O_12_(COO)_16_] clusters.^78^ The structural details of PCN-415 provided important insights on the mechanism of its photocatalytic properties, and could explain how electrons transfer from the valence bands at the NH_2_- functionalized benzene-1,4-dicarboxylic acid (BCD) to the conduction bands of the Ti(iv) cations.

**Fig. 10:**
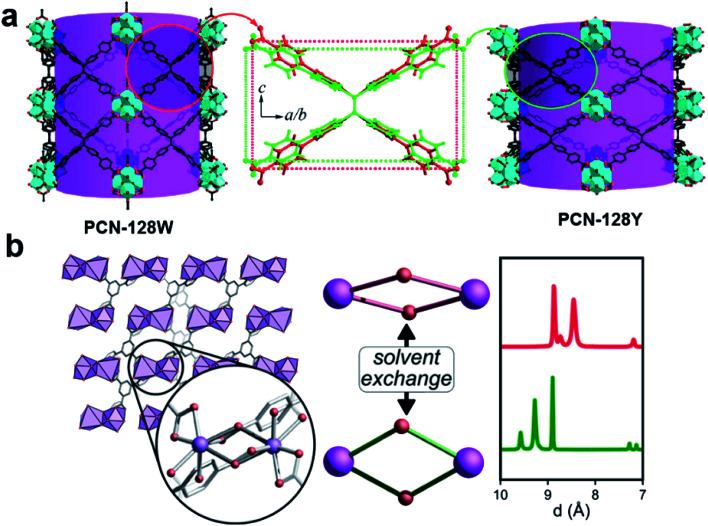
Illustration of structural changes in flexible MOFs (a) PCN-128 ^74^ and (b) SU-100,^81^ whose structures are revealed by 3DED. Reproduced from ref. 74 and 81 with permission from the American Chemical Society, copyright 2015 and 2019.

Cooperative structural changes are one of the unique properties of MOFs. The structural transformability can be triggered by the host–guest interactions as well as by external stimuli.^86,87^ However, the structural changes may lead to loss of crystallinity that prevents the structural analysis by SCXRD. 3DED was applied to gain an insight into the structural changes of MOFs at atomic levels, and to understand their properties and functionalities. The first example of novel flexible MOFs studied by 3DED was PCN-128,^74^ which is built from 8-connected Zr_6_ clusters and a chromatic linker 4′,4′′′,4′′′′′,4′′′′′′′-(ethene-1,1,2,2-tetrayl)tetrakis-(([1,1′-biphenyl]-4-carboxylic acid)) (H_4_ETTC). PCN-128 exhibits a piezofluorochromic behavior, with reversible color changes from white (PCN-128W) to yellow (PCN-128Y) upon the change of the exterior or interior pressures. RED revealed that the color change was associated with the change of the bond angles within the rectangular ETTC ligand, making PCN-128W a microscissor lift. The flexible structure led to a bathochromic shift in luminescence, which is a promising property for applications in photocatalysis and sensing (Fig. 10a). Another flexible MOF SU-100, with a composition [Bi(BPT)]·2MeOH (BPT = biphenyl-3,4,5-tricarboxylate), was solved by cRED.^81^ SU-100 exhibits a solvent-dependent flexibility and undergoes reversible volume changes. Different from most flexible MOFs, such as PCN-128 where the flexibility occurs through conformational changes of organic linkers, the breathing phenomenon in SU-100 was associated with its inorganic building unit, a Bi_2_O_12_ dimer, through the changes of the O–Bi–O angles (Fig. 10b).

Beyond the framework structures, 3DED has also shown to be effective in studying host–guest interactions in MOF nanocrystals. The structure of Co-CAU-36, which is linked through Ni-4-tetraphosphonophenylporphyrins (Ni-H_4_TPPP^4−^), was *ab initio* determined by merging eight high-resolution cRED datasets (0.83–1.00 Å).^41^ Importantly, to reduce the beam damage and prevent the removal of guest molecules by vacuum, the sample was cooled at 96 K before being transferred to the TEM. The positions of all non-H atoms in the framework as well as most atoms of the guest solvent 1,4-diazabicyclo[2.2.2]octane (DABCO) and H_2_O molecules in the pores could be located directly from the structure solution using SIR-2014. The remaining atoms were found from subsequent structure refinements. Furthermore, strong hydrogen bonds were identified between the solvent molecules and the framework. It is worth noting that the strong hydrogen bonds are essential to keep the guest molecules stable and ordered in the pores. Importantly, the structural model could be obtained independently from each of the eight cRED datasets. The atomic positions only deviate on average by 0.03(2) Å, for the framework atoms, 0.10(6) Å for the DABCO molecules and by 0.16(11) Å for the water molecules. This indicates a high reproducibility of structure determination by 3DED.

### Low crystalline MOFs

In many cases, it is challenging to grow MOF crystals with high enough crystallinity to obtain sufficiently high-resolution 3DED data (higher than 1.3 Å) required for *ab initio* structure solution. Nevertheless, key structural information including unit cell dimensions and space groups can often be obtained from low-resolution 3DED data. In combination with the chemical information of the building units as well as possible topologies, an initial structural model can be derived using real space approaches such as modeling and simulated annealing. This strategy has successfully been applied to several important MOFs, such as CAU-7,^88^ [Hf_6_O_4_(OH)_4_(HCO_2_)_6_(carboxylate)_6_],^89^ PCN-777,^90^ PCN-333,^91^ UU-100,^92^ and Cu-kag.^93^ It is worth noting that low-resolution 3DED data contain a limited number of reflections, which may not be enough to refine the structural model. Thus, it is important to check whether the intensities calculated from the model agree with the observed 3DED and PXRD data. The structural model should also be validated by other characterization techniques.

**Fig. 11:**
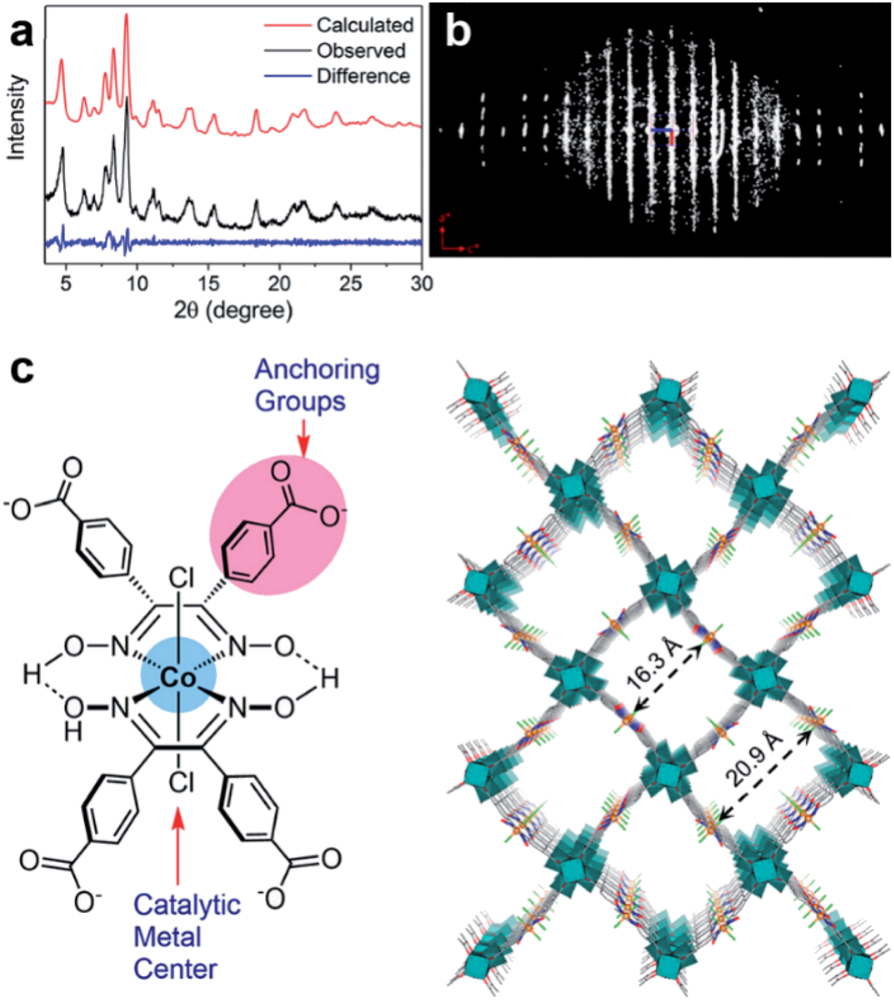
(a) Pawley fitting profiles of the PXRD pattern of UU-100 (*λ* = 1.5418 Å). (b) 3D reciprocal lattice of UU-100 reconstructed from 3DED data. (c) Structure of the cobaloxime linker, and the structural model of UU-100 showing open reticular pore structures.^92^ Reproduced from ref. 92 with permission from the American Chemical Society, copyright 2019.

The importance of 3DED for structure determination of low crystalline MOFs is demonstrated on UU-100,^92^ a Zr-MOF synthesized using a widely studied molecular hydrogen evolution catalyst (HEC) cobaloxime as the organic linker and Zr_6_ cluster as the inorganic building unit. The PXRD pattern shows broad peaks, indicating a low crystallinity of UU-100 (Fig. 11a). The cRED data contain both sharp spots and streaks, indicating the presence of structural disorders (Fig. 11b). Although the unit cell parameters and space group could be determined directly from the cRED data, the data resolution was too low (*ca.* 2 Å along the c*-axis and 4 Å along a*- and b*-axes) for *ab initio* structure solution by direct methods. Instead, the Patterson method was applied to locate the positions of strongly scattering Zr_6_ clusters. Consequently, the structural model of UU-100 was obtained based on the location of the Zr_6_ clusters, the unit cell dimensions, the space group, and the chemical linkage information (Fig. 11c). The PXRD pattern was used to further confirm the unit cell and space group (Fig. 11a), while simulated ED patterns were compared to the experimental patterns to confirm the structural model. The structure revealed the spatial proximity of the redox-active sites, which promotes high electron hopping rates, and is crucial for applying UU-100 as electrocatalysts.

Obtaining structural information is particularly important for mesoporous MOFs, whose large structures often result in low crystallinity and small crystal sizes. Two highly stable mesoporous MOFs, PCN-777 and PCN-333 are among such examples.^90,91^ Both PCN-777 and PCN-333 were designed and synthesized based on reticular chemistry. The same 4,4′,4′′-*s*-triazine-2,4,6-triyl-tribenzoate (TATB) was used as the organic linker, while different metal clusters, Zr_6_ clusters for PCN-777 and Al_3_ clusters for PCN-333, were used as inorganic building units. Their unit cells and space groups were determined from the RED data, as *a* = 55.57 Å and *F*4̄3*m* for PCN-777, and *a* = 126.42 Å and *Fd*3̄*m* for PCN-333. The structural models were obtained based on the possible topologies that are compatible with the building units. The revealed large cavities, 3.8 nm for PCN-777 and 1.1, 3.4 and 5.5 nm for PCN-333, were further utilized to accommodate large molecules and enzymes for catalysis. With an improved data collection method such as cRED, it is now possible to obtain high-resolution data and perform *ab initio* structure determination of mesoporous MOFs, as demonstrated recently on mesoporous Ni-MOF BUT-33^94^ and Zr-MOF CAU-45.^95^

## Application of 3DED on COFs

Growing sufficiently large single crystals (>5 μm) is particularly difficult for COFs.^96^ The majority of COF structures have been determined by analyzing PXRD data, and combined with other characterization methods, such as NMR, IR, *etc.*^97^ Compared to PXRD data of COFs, which often exhibit only a few and broad low-resolution peaks, 3DED offers much more diffraction data with higher resolution. Therefore, it is much easier to obtain unambiguous unit cells and structure solutions from 3DED data. Atomic structures of several novel COF nanocrystals have been determined by 3DED.^98–101^

**Fig. 12:**
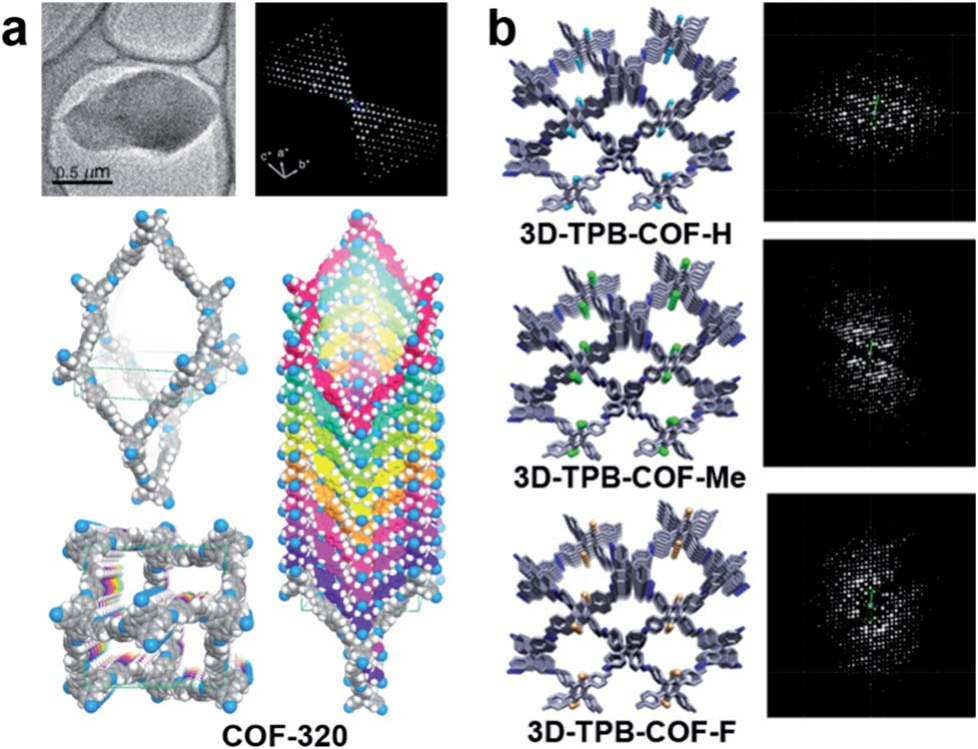
(a) COF-320, with different structures at 298 K and 89 K, which were both determined by RED.^98^ (b) A series of 3D-TPB-COFs, whose structures were determined by cRED.^99^ Reproduced from ref. 98 and 99 with permission from the American Chemical Society, and the John Wiley & Sons, Inc., respectively copyright 2013 and 2019.

The first single crystal COF structure determined by 3DED was COF-320 (Fig. 12a).^98^ RED data were collected at low temperature (LT, 89 K) to reduce the beam damage and improve the data quality. The structure of COF-320 with 9-fold interpenetration was directly determined, with tetra-(4-anilyl)methane (TAM) and 4,4′-biphenyldialdehyde (BPDA) connected in a dia framework. The resolution of RED data was lower when data were acquired at room temperature (RT). Nevertheless, based on the key structural information extracted from 3DED, it was possible to model the structure. The RT structure of COF-320 has the same connectivity and degree of interpenetration as the LT structure, but with different pore sizes.

Continuous rotation electron diffraction data collection methods, such as cRED, can provide higher data resolution than RED. This has been demonstrated on three isostructural 3D-TPB-COFs built by tetra(*p*-aminophenyl)methane (TAPM) and 1,2,4,5-tetraphenylbenzene (TPB) with -H, -Me, or -F substituents, respectively. cRED data of all three COFs have a resolution better than 1.0 Å, allowing an accurate *ab initio* structure determination where all non-H atoms can be found directly.^99^ The structure solutions show that the 3D-TPB-COFs have the same topology with a five-fold interpenetrated pts net (Fig. 12b).

## Conclusions and outlook

Porous crystalline materials have attracted great interest in various fields due to their versatile physical and chemical properties. In this Minireview, we describe the development and application of 3DED techniques for structural analysis of zeolites, MOFs and COFs from nano- and submicron-sized crystals, which are too small for conventional SCXRD analysis. We describe how the detailed atomic structures obtained by 3DED reveal the key structural features that affect the properties in zeolites, such as pore size and dimensionality that affect mass transport, cavities that accommodate reaction intermediates, as well as the locations of heteroatoms that facilitate functionality and catalysis. For MOFs and COFs, besides the porosity, atomic arrangements define the electronic states, which affect the bandgap, charge separation ability, as well as chemical properties such as framework flexibility, and guest–host interaction. Notably, new insights into the chemistry of porous crystalline materials can be revealed, and new strategies for design and targeted synthesis of novel materials can be uncovered by 3DED.

Despite the meritorious examples, development and application of 3DED techniques are still in the dawn. More efforts are needed to further develop 3DED techniques and explore their applications in porous materials. While the demands on TEM expertise have been considerably reduced with the establishment of practical protocols for 3DED data collection, knowledge on both TEM and crystallography is still required to collect and process 3DED data. Full automation of the data collection and processing procedures would be beneficial for non-experts to use 3DED for structural analysis. SerialRED^43^ has been developed as a prototype for such purposes, yet many improvements are desirable. Although 3DED can provide accurate structural information regarding the frameworks, as well as guest molecules, dynamical effects caused by multiple scattering are still a serious concern. To uncover more structural details, such as locations of OSDAs, doped heteroatoms, and hydrogen atoms,^42^ as well as absolute structures,^102^ multiple scattering needs to be properly addressed. Considering the importance of dynamic behaviors in porous crystalline materials, further development on *in situ* 3DED is crucial to gain the fundamental understanding on this aspect. Another remaining challenge is to prevent the loss of crystallinity caused by vacuum and electron beam damage.

In conclusion, structural studies of porous crystalline materials at atomic levels are indispensable to fundamentally understand their physical and chemical properties, which in turn is important for the development of new materials. As TEMs are widely available in laboratories around the world compared to synchrotron facilities, we foresee that the impacts of 3DED for structural analysis will continue to increase, which will certainly accelerate research in the fields of porous materials.

## Conflicts of interest

There are no conflicts to declare.

## Supplementary Material

SC-012-D0SC05731B-s001

SC-012-D0SC05731B-s002
